# UV-C Light: A Promising Preservation Technology for Vegetable-Based Nonsolid Food Products

**DOI:** 10.3390/foods12173227

**Published:** 2023-08-27

**Authors:** Rose Daphnee Tchonkouang, Alexandre R. Lima, Andreia C. Quintino, Nathana L. Cristofoli, Margarida C. Vieira

**Affiliations:** 1 MED—Mediterranean Institute for Agriculture, Environment and Development and CHANGE—Global Change and Sustainability Institute, Faculty of Sciences and Technology, Universidade do Algarve, Campus de Gambelas, 8005-139 Faro, Portugal; rdtchonkouang@ualg.pt (R.D.T.); carlima@ualg.pt (A.R.L.); anquintino@ualg.pt (A.C.Q.); nlcristofoli@ualg.pt (N.L.C.); 2 Department of Food Engineering, High Institute of Engineering, Universidade do Algarve, Campus da Penha, 8000-139 Faro, Portugal

**Keywords:** fruits, vegetables, plant-based, minimally processed, UV-C treatment, bioactive, nutrients, nutrition security

## Abstract

A variety of bioactive substances present in fruit- and vegetable-processed products have health-promoting properties. The consumption of nutrient-rich plant-based products is essential to address undernutrition and micronutrient deficiencies. Preservation is paramount in manufacturing plant-based nonsolid foods such as juices, purees, and sauces. Thermal processing has been widely used to preserve fruit- and vegetable-based products by reducing enzymatic and microbial activities, thereby ensuring safety and prolonged shelf life. However, the nutritional value of products is compromised due to the deleterious effects of thermal treatments on essential nutrients and bioactive compounds. To prevent the loss of nutrients associated with thermal treatment, alternative technologies are being researched extensively. In studies conducted on nonsolid food, UV-C treatment has been proven to preserve quality and minimize nutrient degradation. This review compiles information on the use of UV-C technology in preserving the nutritional attributes of nonsolid foods derived from fruit and vegetables. The legislation, market potential, consumer acceptance, and limitations of UV-C are reviewed.

## 1. Introduction

Millions of people worldwide are undernourished and affected by “hidden hunger”, which is caused by a lack of essential minerals and micronutrients. Food items need to contain enough nutrients, whether processed or unprocessed, so that these nutrients can be significant contributors to food and nutrition security [1,2]. The majority of consumers view food safety as being of the utmost importance [3]. On the other hand, they are increasingly aware of nutrient uptake and seek to consume more foods that will benefit their health, well-being, and nutritional status. The increased consumption of fruit- and vegetable-based products has been motivated by the potential health benefits based on the significant amounts of vitamins, nutrients, and bioactive compounds contained in these products [4]. Several fruit- and vegetable-based products are preferred in their fresh state. However, they have a high perishability and a short shelf life. This limits the amount of time for which they are available and safe for consumption. Processing techniques can increase food choices while increasing the length of time before a food product becomes unfit for consumption. In the manufacture of processed foods, the use of preservation strategies is unavoidable in suppressing microbial or enzymatic and nonenzymatic spoilage, and therefore achieve an extended shelf life [5].

Thermal processing has historically been one of the most extensively used and approved methods to prevent foodborne illnesses and ensure food safety through the inactivation of spoilage enzymes and the destruction of microbial contaminants (pathogenic and spoilage) in foods and beverages [6]. The intensity of the heat treatment is dependent on the combination of temperature and treatment duration. From a microbiological perspective, intense heat treatment is preferable, but the employment of excessively high temperatures during prolonged times (severe heat treatments) can have deleterious consequences on the flavor, taste, and nutritive quality. Hence, a food product may be free of contaminants, comply with food safety standards, and still be nutritionally poor [7]. For instance, severe heat treatments degrade several heat-labile vitamins (e.g., vitamins A and C, and thiamin) and decrease the biological value (BV) of proteins by denaturing them and reducing their digestibility and bioavailability. The significance of nutrient degradation on nutrition security is determined by the eating habits and consumption frequency of a certain kind of food in the diet. Loss of nutritional value is thus more significant when there is a decrease in nutrients in nutritionally-rich and highly consumed food items that are sources of nutrients for a large share of the population than in foods that are either consumed in low quantities or have low nutritional contents [8,9].

Novel food processing methods are under investigation to address the loss of nutritional value due to thermal preservation [10]. Food processors and scientists have been exploring more effective low-temperature technologies that enable high-quality retention to deliver safe food products with acceptable organoleptic and rich nutritional profiles [7]. Nonthermal processing methods have been employed and among these, ultraviolet irradiation holds great promise as a food preservation technique for pathogen reduction and to minimize nutritional losses observed in heat-processed foods [11,12]. Ultraviolet radiation is divided into four categories in terms of wavelength range: UV-A (315–400 nm), UV-B (280–315 nm), UV-C (200–280 nm), and vacuum-UV (100–200 nm) [13]. The UV-C range possesses great antimicrobial effectiveness, which makes it useful for ensuring the microbial safety of foods. The genetic material (DNA or RNA) of microbes strongly absorbs UV photons within the UV-C range, with a wavelength around 260–265 nm corresponding to maximal UV absorption [14]. The preferred alternative pasteurization and shelf life extension method for beverages for the past two decades has been UV-C radiation at 253.7 nm [15]. UV-C irradiation causes damage to the nucleic acids of microorganisms, mainly due to the formation of dimers of pyrimidine bases between adjacent pyrimidines in a DNA strand, which prevents microbial replication and ultimately leads to cell death [16,17].

UV-C is a nontoxic and noninvasive method with numerous advantages that include the absence of chemical residues, it produces no waste, is cost-effective (low installation and maintenance cost), simple to implement, eco-friendly, has low energy consumption, minimal impact on nutritional quality and organoleptic parameters, and good consumer perception [11,15,18,19]. The primary drawback of this technology is the poor penetration depth of UV-C, which limits its antibacterial efficacy [20]. The microbial inactivation efficiency of UV-C is dependent on several factors like the UV-C dose (UV-C fluence), uniformity of UV-C dose distribution, UV-C sensitivity of the target microbial cells, the ability of the microorganisms to repair UV-induced damage, the physicochemical properties of the treated product (e.g., viscosity, density, soluble and suspended solids), and the optical properties of foods (e.g., transparency, absorption coefficient, scattering) [16,21,22,23]. This poses difficulties in the design of UV-C food treatment devices and for laboratory tests (experiments) that must guarantee a defined and consistent UV-C delivery while ensuring that all of the food surfaces are exposed to the UV-C illumination [22]. This paper provides a review of the impacts induced by ultraviolet pasteurization and ultraviolet combined pasteurization on the composition of nutrients and bioactive compounds on UV-C treated products. The regulatory standards, associated cost, consumer perception, and limitations of this emerging technology are equally discussed.

## 2. UV-C Light: Principles and Mechanisms of Germicidal Action

The principle behind UV-C light’s germicidal action is based on its ability to damage the DNA or RNA of microorganisms such as bacteria, viruses, and fungi through interaction between the UV photons and the genetic material of these microorganisms [21]. When UV-C light penetrates the cell wall of a microorganism, it is absorbed by the DNA or RNA inside the cell. This disrupts the genetic material, which can lead to the formation of new bonds or the breakage of existing ones. This alteration results in photodimerization, where two adjacent bases in the DNA/RNA sequence bind together. This genetic damage disrupts the affected cells’ ability to replicate, rendering them unable to cause infection or pose a threat [24].

**Figure 1:**
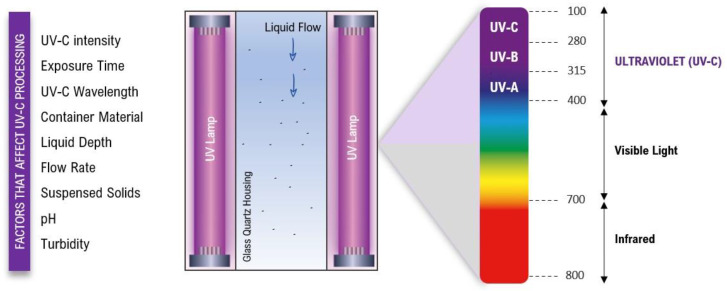
Reactor chamber of UV-C processing for fluid food and the main factors that influence the process.

The mechanism of UV-C germicidal action involves several factors including the light intensity, exposure time, and the type of microorganism being targeted, which can vary depending on the specific application [21,25]. Furthermore, the germicidal effectiveness of UV-C light as a disinfectant is based on the dose–response relationship, microbial susceptibility, and the optical properties of the food matrices or treated surfaces [26,27]. In Figure 1, the main factors that affect the success of UV-C processing are presented as well as a general representation of the reactor chamber.

It is important to note that these factors are interconnected and should be considered collectively during the design and implementation of UV-C treatment processes for developing shelf-stable food. Higher intensity levels of UV-C radiation generally lead to better microbial inactivation [28]. However, the duration of UV-C exposure should be carefully selected to achieve microbial reduction without compromising food composition and quality [29,30]. Furthermore, the choice of the UV-C wavelength should be based on the target microorganisms and the food product [24]. The material of the product’s container can affect UV-C treatment, with transparent materials allowing for better penetration; the depth of the liquid and flow rate through the UV-C system should be considered for uniform exposure [31,32]. At the same time, suspended solids can reduce the effectiveness of UV-C treatment [25,33]. The pH and turbidity of the liquid also impact treatment efficiency, and maintaining optimal ranges enhances the effectiveness of UV-C treatment [25]. From the understanding of these principles, UV-C light technology has been used effectively not only for disinfection and sterilization in various applications such as healthcare settings and water treatment, but also in the food industry and more recently as a neutralizing agent of the infectivity of SARS-CoV-2 [22,34,35,36,37,38]. Some factors that influence UV-C efficacy are described below.

### 2.1. Dose–Response Relationship

The dose-response relationship of UV-C light germicidal action follows a pattern where the effectiveness of killing microorganisms increases with higher doses or intensities of UV-C light [39,40]. At lower doses, the light exposure may not be sufficient to cause significant damage to the microorganisms, allowing some of them to survive or repair the damage [12,40,41]. As the dose of UV-C light increases, the likelihood of DNA and RNA damage also increases, leading to a higher rate of microorganism inactivation [42].

It is important to note that there is an optimal range of UV-C light intensity for germicidal action. The sensitivity of microbes to UV light varies depending on the wavelength [21]. However, the strong absorption of ultraviolet light by water at wavelengths below 230 nm is a limiting factor for the germicidal effect. Beyond this wavelength, increasing the dose may not significantly enhance the killing efficacy and may even result in diminishing returns. Additionally, excessively high doses of UV-C light can harm human health and damage materials or surfaces [37,43]. In this sense, it is crucial to use UV-C light within safe and recommended exposure limits to balance its germicidal efficacy with potential risks.

### 2.2. Microbial Susceptibility

The susceptibility of microorganisms to UV-C light varies depending on their structure and genetic makeup [33]. UV susceptibility of microorganisms can differ considerably due to differences in cellular elements like cell wall thickness, composition, nucleic acid structure, type of proteins within the cell, photoproducts, the physiological condition of the microbe, and the cell’s capacity for repairing damage caused by ultraviolet radiation [19]. However, it is worth mentioning that the effectiveness of UV-C light as a microbial inactivation method depends on other factors including the food matrix [44], exposure time, distance from the UV-C source, and the presence of any physical barriers or shadows that may shield microorganisms from direct UV-C exposure [42]. Different microorganisms have varying levels of sensitivity to UV-C-induced DNA/RNA damage. In this sense, viruses with RNA genomes are more susceptible to UV-C light than viruses with DNA genomes [45]. Another important factor is the cell wall structure. Microorganisms with more robust and resistant cell walls may be more resistant to germicidal UV-C light. Viruses and fungi, on the other hand, may be more susceptible to UV-C light due to their fragile cell walls. Gram-negative bacteria, in general, are more sensitive to UV-C light than Gram-positive bacteria due to their thinner cell walls [46]. The efficacy of UV-C microbial inactivation greatly depends on the treated food. Opaque and turbid nonsolid food matrices are more challenging to treat compared to transparent food substrates. This is because the turbidity and presence of suspended solids in nontransparent liquids confer protection to microorganisms by scattering or absorbing the radiation before it reaches them [44].

### 2.3. Optical Properties of Surfaces

The optical properties of surfaces refer to how they interact with light. These properties can include the reflection, absorption, transmission, and scattering of light [21,32]. When it comes to UV-C light, the optical properties of surfaces that host microorganisms can affect the effectiveness of UV-C light. For example, surfaces that are rough or uneven may scatter UV-C light, potentially reducing the intensity of UV-C radiation in a particular direction [32], and if they are porous, UV-C light can be absorbed. Reflective surfaces can also scatter and absorb UV-C light [22,37]. When compared to smooth surfaces, some of these surfaces require roughly two orders of magnitude greater UV-C doses to adequately inactivate microorganisms [37,47]. Normally, light transmission refers to the passage of UV-C light through materials. Materials like certain types of glass can allow UV-C light to pass through with minimal attenuation, while others may block or attenuate UV-C light, reducing its transmission [31,32].

## 3. Current Applications of UV-C Light in the Food Industry

The recent consumer demands for safe food with high-quality nutritional (e.g., vitamins, protein) and sensory (mainly color, flavor, and texture) attributes have challenged the scientific community and the food industry to develop and implement nonthermal technologies to process/manufacture foods while minimizing the changes to these attributes [48,49,50]. In this sense, UV-C light has been a promising technology for improving food safety and reducing the risk of foodborne illnesses in the food industry [21,33]. In the last decades, the food industry has used this versatile tool for surface decontamination, air and water treatment, to prevent the spread of microorganisms, and ensure food safety and preservation.

### 3.1. Air Purification and Surface Disinfection

UV-C light is used to purify air in food processing facilities. UV-C lamps can be installed in air handling units to sterilize the air as it circulates through the facility, reducing the risk of airborne contamination [35]. Air disinfection can be accomplished by irradiating only the upper parts of the room or by irradiating the entire air, either in an empty room or using an air conditioner [51]. UV-C light is also used to disinfect surfaces following routine cleaning procedures in food processing facilities including food preparation areas, packaging areas, and equipment. UV-C light can effectively kill bacteria, viruses, and other microorganisms that may contaminate surfaces and cause foodborne illness [35,52,53]. Low-pressure mercury lamps are ideal for controlling surface microorganisms in the food industry, since 90% of the emitted light is at a 253.7 nm wavelength [54].

### 3.2. Water Treatment and Food Preservation

UV-C light can be used to sanitize water used in food processing and production as well as help prevent the growth of harmful bacteria and other microorganisms in municipal water supply systems [53,55]. Additionally, UV-C light has been used to extend the shelf life of fresh, minimally processed, and liquid foods by reducing the microbial load and helping to prevent spoilage [12,56,57,58,59,60].

### 3.3. Retention of Bioactive Compounds

While UV-C light technology is commonly used for its antimicrobial properties in the food industry, there is also research indicating that it can be used to improve and/or preserve the nutritional properties of fruit and vegetables [33,60,61,62,63]. When exposed to UV-C light, certain compounds in foods can be activated or transformed, resulting in the production of bioactive compounds that may have health benefits [64,65,66]. Bhat and Stamminger (2014) reported that exposure to UV-C light has been shown to increase the levels of phenolic compounds and antioxidant activity in strawberry juice [48]. In the same way, UV-C light exposure has been shown to increase the levels of certain phytochemicals in plant produce [67].

Győrfi et al. (2011) identified the capacity of UV-C light to increase the production of vitamin D in mushrooms. When exposed to UV-C light, the ergosterol in mushrooms is converted to vitamin D2, increasing the vitamin D content [68]. Overall, UV-C light can be a useful tool for producing bioactive compounds in foods, which can enhance their nutritional value and potential health benefits. However, it is important to carefully evaluate the safety and efficacy of these compounds before incorporating them into food products.

## 4. Ultraviolet Light for the Preservation of Fruit- and Vegetable-Based Nonsolid Foods

**Figure 2:**
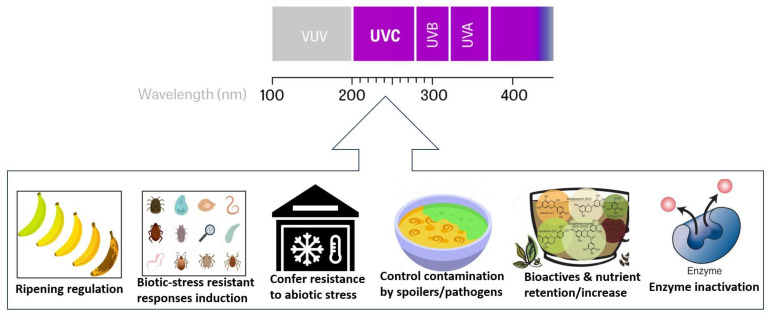
Desirable Outcomes of UV-C from direct application on fruits and vegetables.

There is a growing demand for fresh foods such as fruits and vegetables that are ready to eat, nutritious, safe, free of additives, and can be included in a healthy diet. However, the convenience and attractiveness of these high-in-demand fresh foods and beverages are affected by rapid spoilage and short shelf life due to changes that can be physical, chemical, microbiological, and enzymatic [69]. Frequent outbreaks of foodborne pathogens are associated with fresh produce and fruit juices. The addition of chemical preservatives to liquid foods and beverages to extend their shelf life and protect against foodborne pathogens is eliciting negative consumer acceptance. UV irradiation has been used mainly for microbial load reduction in liquid foods and beverages such as milk, juices, ciders, liquid eggs, beverages, and honey [19]. In terms of plant-based products, UV has been applied as a nonthermal method to improve the safety and shelf life of products such as vegetables, fruits, cold-pressed juices, plant-derived milk alternatives, and nectars [25,70,71,72], with a demonstrated ability in inactivating a wide range of microbial pathogens (e.g., bacteria, fungi, yeasts, molds, and viruses) [25]. Figure 2 demonstrates some positive outcomes from the application of UV-C in the fruit and vegetable sector.

### 4.1. Microbial Inactivating Effect

Liquid food products have a diverse range of physical (e.g., viscosity and density), chemical, and optical properties. Each group of properties must be properly evaluated to design the preservation process and optimize the performance of the UV reactor. The physical properties influence the effectiveness of the fluid momentum transfer and the flow pattern. Optical properties are the main factors affecting UV light transmission and hence microbial inactivation in liquid foods. Chemical composition, pH, dissolved solids (°Brix), and water activity are considered obstacles that can modify the effectiveness of UV inactivation [21]. The sensitivity of microorganisms to ultraviolet radiation varies significantly due to differences in cellular components such as cell wall structure, thickness, composition, nucleic acid structure, type of cellular proteins, photoproducts, physiological state of the microorganism, and the ability of the cell to repair the damage caused by ultraviolet radiation [19].

Numerous applications of UV treatment in plant-based nonsolid foods have been recorded. In a study conducted by Caminiti et al. (2010), reconstituted apple juice was exposed to UV light in a continuous laboratory scale system with doses ranging from 2.66 to 53.10 J/cm^2^, altering the exposure time. The treated and untreated juices were evaluated for microbial count and selected physical and chemical attributes. Microbiological analysis was performed by inoculating apple juice with *Escherichia coli* K12 and *Listeria innocua* and the bacterial count was estimated before and after processing. Overall, this study demonstrated that UV technology applied for short periods can represent a valid alternative to the heat treatment of reconstituted apple juice by reducing *E. coli* and *L. innocua* counts below the detection limits [73]. Mango nectar was UV-C-treated at varying flow rates of 0.073 and 0.451 L/min and analyzed for yeast and total microbial counts during storage at 3 °C. The highest log reduction obtained from the UV-C exposure at 0.451 L/min for 30 min was 2.71 CFU/mL for the total microbial count and 2.94 CFU/mL for the yeast count [71]. Table 1 shows some examples of how UV-C light has been used to reduce the microbial load in nonsolid fruit- and vegetable-based foods.

### 4.2. Preservation of the Biological Activities of Foods

Regarding the use of UV treatments, preventing the loss of nutritional quality and bioactive compounds is the aspect that has received the most attention, after microbial control. Bioactive compounds are extra-nutritional constituents, available mainly in fruits and vegetables, that confer additional health benefits to humans [80]. A few examples of bioactive components include phytosterols, phytoestrogens, glucosinolates, polyphenols, taurine, carotenoids, flavonoids, carnitine, choline, coenzyme Q, and dithiolthiones. Vitamins and minerals possess pharmacological activity and can also be classified as bioactive compounds for this reason. The majority of biologically active compounds contain antimicrobial, anticarcinogenic, anti-inflammatory, and antioxidant activities [81]. In contrast to the major nutrients, bioactive substances are neither officially recommended nor listed by governmental organizations [82].

The antioxidant potential is one of the most important properties that protects against harmful free radicals known to contribute to the occurrence of chronic conditions such as cancer and age-related degenerative diseases. Moreover, antioxidants are a good predictor of the biological activity and health-protective properties of foods since they are known to inhibit oxidative damage in organisms [51,80,83]. Living plant tissues (e.g., peppers and blueberries) frequently experience hermetic reactions from UV-C light, which stimulates the production of secondary metabolites and raises their antioxidant capacity [84]. Likewise, UV-C light can induce the formation of phenolic compounds, which have gained popularity as anticancer agents [85,86]. On the other hand, UV-C exposure has been shown to cause oxidation in several fruit and vegetable juices and purees. For example, after being exposed to UV-C radiation, the total phenolic and vitamin C content of apple juice dropped considerably, and this deterioration was accelerated in clarified apple juice. Clarification increases light transmittance and removes the intrinsic protective compounds enhancing UV-C’s effect on food components. Additionally, a decrease in the antioxidant activity of UV-C treated horchata beverage against DPPH radicals has been reported [84].

Pala and Toklucu (2011) conducted a study where they exposed apple juice to UV radiation to preserve the main quality characteristics such as anthocyanins, polymeric color, antioxidant activity, and total phenol content. The obtained results were compared with the control (i.e., the untreated juice), and a better preservation of the studied parameters was obtained with UV-C [87]. After being exposed to UV-C for durations ranging from 5 to 25 min, blueberry and raspberry nectars were reported to contain more total monomeric anthocyanins [88]. Table 2 shows some examples of how UV-C light has been used to produce, increase, or retain bioactive compounds in liquid foods, but the benefits are proven to be extended to solid foods.

In summary, the proper application of UV-C as a preservation method can be beneficial in terms of food composition. However, it is also possible that UV-C (particularly prolonged UV-C exposure) may have an unfavorable effect on the food’s nutritional value because a number of photochemical reactions may induce undesirable outcomes such as the production of free radicals, potentially reducing the amount of beneficial components found in food [97].

### 4.3. Endogenous Enzyme Inactivation

The spoilage of fruit- and vegetable-based products is closely linked to the activity of various endogenous enzymes such as browning enzymes (e.g., polyphenol oxidase, peroxidase, and phenylalanine ammonia-lyase) and ripening or cell wall degrading enzymes (e.g., pectin methyl-esterase) [98]. Controlling the activity of these enzymes is critical because they remain active after processing, negatively affecting the quality, nutritional content, and shelf life [99,100]. In Table 3, data on enzyme inactivation using ultraviolet technology is provided, reporting the data sources.

### 4.4. Modeling the Kinetics of Preservation of Bioactive Compounds and Nutrients

Kinetic models are often used for objective assessment and economic evaluation of food safety. Kinetic modeling can also be used to predict the influence of processing on critical quality parameters. Knowledge of the kinetics of food quality degradation including reaction order and half-life is critical for predicting the food quality loss during storage and preservation processes. One of the crucial factors to consider in processing is compositional changes due to nutrient loss. Therefore, kinetic studies are essential to minimize unwanted variation and optimize the quality of specific food [106].

Considering that the kinetics of nutrient changes in fruits and vegetables usually follows either a zero-order or a first-order model, Equations (1)–(4) [107] are

(1) $$\frac{dP}{dt}=-k\;{\left(P\right)}^{n}$$

where *k* is the nutrient rate constant, *n* is the reaction order, and *P* is the parameter of the nutrient to be estimated with a variable time, *t.* In the zero-order reaction kinetic model, the nutrient parameters are usually independent of the reaction rate, as shown in Equation (2):

(2) $$-\frac{dP}{dt}=k$$

Integrating Equation (2) yields Equation (3):

(3) $$P=P_{0}\pm kt$$

where *P*_0_ is the value of the nutrient parameter at time zero and ± would typically signify the increase or degradation of the nutrient parameter. When the reaction rate is dependent on the nutrient parameter, the solution of a first-order reaction rate Equation (1) is expressed by Equation (4):

(4) $$P=P_{0}\exp\left(\pm kt\right)$$

### 4.5. The Combined Use of UV-C with Other Preservation Technologies

The limited microbial lethality of UV light in food matrices with a high absorption coefficient and turbidity such as nontransparent liquid foods promoted the emergence of combined processes and hurdle technology. UV technology can be combined with conventional and other nonthermal processes to increase the lethal effects of UV light on microorganisms. The germicidal degree of combined treatments can result from an additive or synergistic effect. Synergistic lethal effects are preferable in the design of combination processes because a specific level of inactivity can be achieved by reducing energy consumption and treatment intensity/severity [108].

Several studies have investigated the effectiveness of UV irradiation treatment in combination with other treatments. In one research study, UV irradiation combined with laser irradiation was effective against *Bacillus cereus* compared to UV irradiation or laser irradiation alone [19]. The combination of ultraviolet-C radiation and ultrasonic technology as a barrier approach provides increased efficiency, cost-effectiveness, and reduced processing time without compromising quality. These are widely accepted and are continually being evaluated as alternatives to conventional thermal techniques for decontaminating fruits, vegetables, and derived products. However, studies in these areas have presented challenges related to quality, safety, limited capacity, and energy cost [109]. According to Gayán et al. (2014), the simultaneous application of UV light with gentle heating, oxidizing agents, or cell membrane fluidizing compounds can result in successful inactivation treatments [110]. The application of UV-C pulsed radiation in combination with thermosonication at 90 °C resulted in a 3-log reduction of the heat-resistant and radiation-resistant cells of the vegetable contaminants, *Enterococcus faecalis* and *Deinococcus radiodurans,* surpassing the industrial requirement of 2-log reduction [12]. Table 4 demonstrates some examples of how UV-C light combined with other treatments has been used to reduce the microbial load in nonsolid fruit- and vegetable-based foods.

## 5. Ultraviolet Reactors for Nonsolid Food Pasteurization

UV-C irradiation in liquid foods can be performed in equipment that either uses batch or continuous operation modes. In batch processing, the product is placed in a glass container within a UV-C irradiation chamber. The product is then irradiated for a predetermined amount of time at a given UV-C dose. Continuous operation mode involves pumping the product into a high-permittivity UV light tube, coiled tube, or jacketed reactor that contains lamps that emit UV-C light. In continuous systems, UV-C exposure is performed for minutes to hours during which the product flows around the lamps with or without recirculation [110,114,115]. Continuous UV-C is preferable for industrial applications because it could present advantages over batch processing such as increased productivity [115].

A variety of UV light sources have been used in UV-disinfection systems including pulsed-light (PL), excimer lamps, low-pressure mercury (LPM), medium-pressure mercury (MPM), low-pressure high output mercury lamp-amalgam type, mercury-free amalgam lamps, and so on [116]. LPM lamps currently serve as radiation sources in the majority of UV-based disinfection systems for the treatment of nonsolid foods and beverages [117]. More recently, ultraviolet light-emitting diodes (UV-LEDs) have been employed in treating juices and beverages in continuous reactors [118]. There are various types of UV-C reactors with various flow patterns. The flow pattern in the reactor has a major impact on the UV-C dose required for the inactivation of undesirable microorganisms. Hence, to maximize the homogeneity of UV-C treatment, it is necessary to enhance the flow conditions [119]. In general, four flow types can be identified as follows: Taylor–Couette flow, Dean–Vortex flow, laminar, and turbulent flow. Various reactor designs or systems are often utilized to achieve the aforementioned flow characteristics [120].

### 5.1. Laminar and Turbulent Flow Reactors

**Figure 3:**
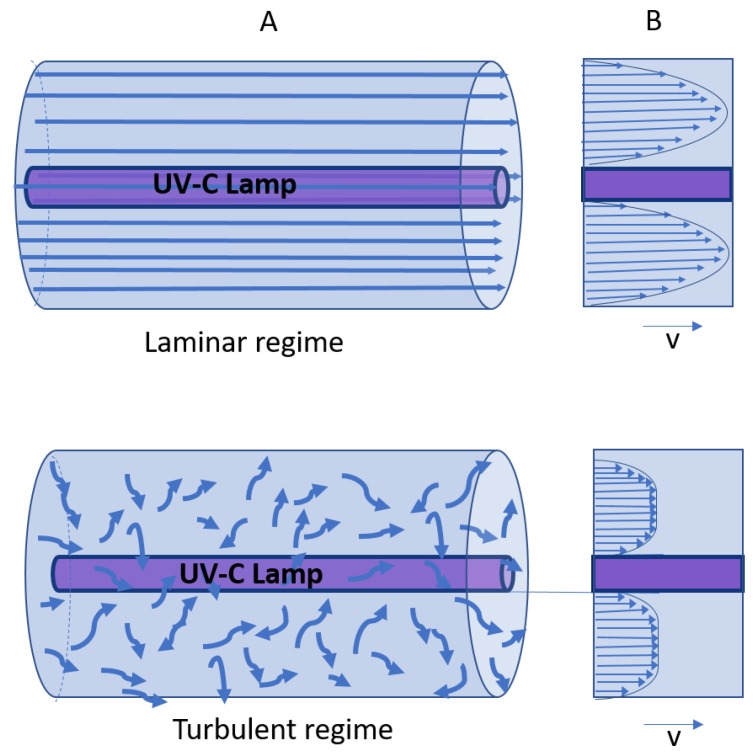
Flow through a UV-C reactor with one lamp inside a pipe. (**A**) 3D view and (**B**) cross section view of the velocity profile for both the laminar and turbulent regimes.

Laminar flow is a flow type where the fluid travels smoothly or through regular paths, as opposed to turbulent flow where the fluid experiences unstable fluctuations and mixing [121]. Figure 3 is a schematic illustration of laminar and turbulent flows.

**Figure 4:**
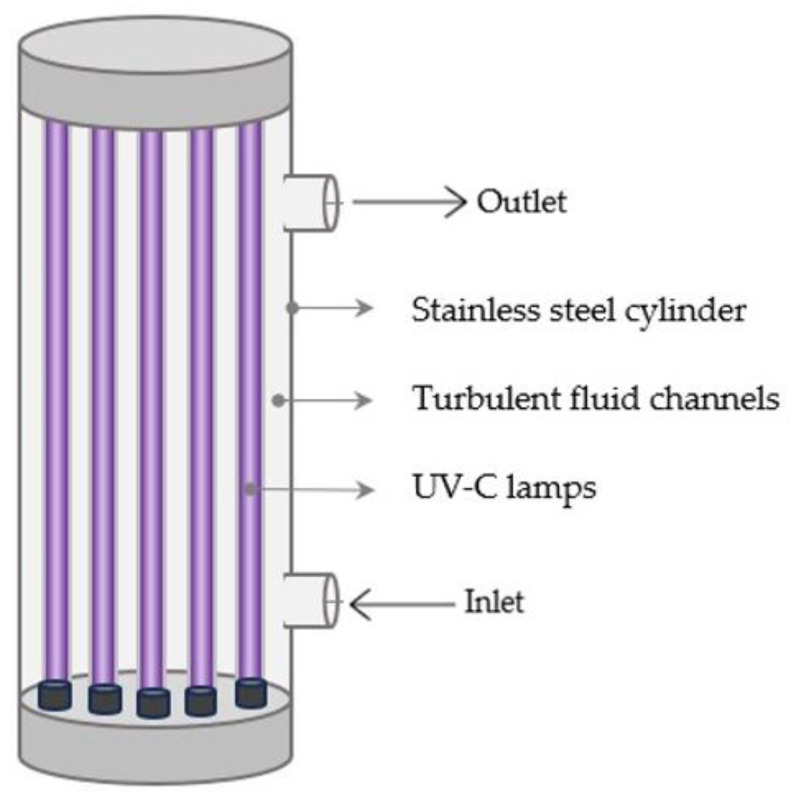
Diagram of a turbulent UV-C reactor.

The low radial mixing in laminar flow systems reduces their efficiency in facilitating a uniform UV dose distribution [122]. Turbulent flow reactors, on the other hand, use higher flow rates to increase turbulence within a UV reactor, thereby enabling close contact between the UV-C light and the product’s constituents during treatment and overcoming product turbidity, which interferes with UV penetration. The turbulent flow mechanism efficiently mixes the fluid, allowing for a more uniform UV-C dose distribution [97,123]. Different types of turbulent flow UV-C reactors have been developed. In the Aquionics UV-C turbulent reactor (Hanovia, Slough, UK), the fluid passes through a cylindrical compartment made of stainless steel in which 12 parallel 42 Watt UV-C low-pressure lamps are housed in a quartz sleeve (Figure 4) [110].

**Figure 5:**
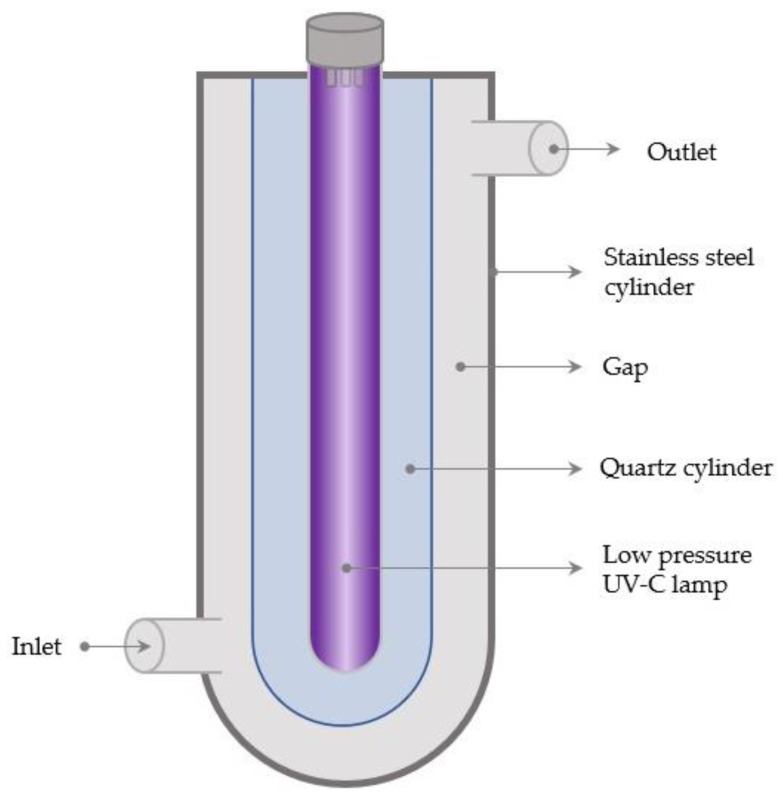
Diagram of a thin film annular UV-C reactor. Flow type can be laminar or turbulent.

Laminar and turbulent flow regimes were previously used in thin film reactors. The intended effect of thin film reactors is to shorten the path of UV radiation to maximize the UV-C radiation delivery to the food or beverage, thus providing a solution to the inadequate penetration of UV photons [124]. Using laminar and turbulent flows in continuous thin film reactors, Koutchma et al. (2004) investigated the effectiveness of UV radiation to inactivate *E. coli* K-12 in apple juice. They observed that the inactivation of *E. coli* in apple juice increased under turbulent flow conditions due to enhanced mixing [125]. In another study, turbulent flow conditions in an ultra-thin film annular reactor produced a better UV dose distribution and higher microbial inactivation rate compared to a laminar flow regime. The microbial inactivation rates were found to increase as the flow rate increased due to greater turbulence intensity [126]. A diagram of a thin film annular reactor is provided in Figure 5. The annular structure is utilized because it is effective for deactivating microorganisms. It is made of a single lamp that is positioned at the center of the reactor. The product flows in the annular gap. The flow in this gap might be turbulent or laminar depending on the flow rates [127].

### 5.2. Taylor–Couette Reactors

**Figure 6:**
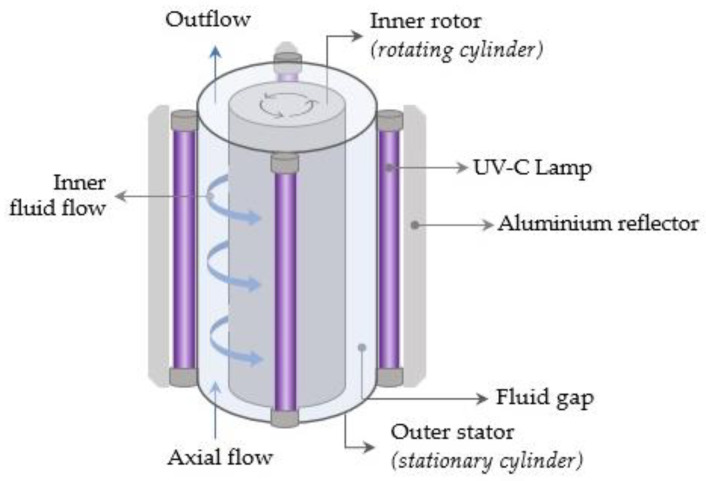
Taylor–Couette UV-C reactor.

The flow between two coaxial cylinders with an inner rotating cylinder is referred to as Taylor–Couette flow [128,129]. As seen in Figure 6, the Taylor–Couette ultraviolet unit is made up of two concentric cylinders: an outer stator (outer stationary cylinder) and an inner rotor (inner rotating cylinder). The fluid product is pumped through the annular space between the cylinders and subjected to UV-C irradiation that originates from lamps positioned around the outer cylinder. The rotation of the inner cylinder creates a Taylor–Couette flow [52]. The vortices produced in the Taylor–Couette UV reactors have the potential to deliver effective radial and axial mixing. Furthermore, the thickness of the fluid boundary layer between the fluid and the UV lamps is minimized, resulting in optimal UV exposure times for the undesired microorganisms and uniform radiation intensities [130]. Several flow regimes can be obtained under different flow and rotation rates in a Taylor–Couette system [128]. Ye et al. (2008) demonstrated that higher log reduction levels of *Escherichia coli* K12 (ATCC 25253) and *Yersinia pseudotuberculosis* could be achieved with laminar Taylor–Couette flow as opposed to turbulent or laminar Poiseuille flow. The authors concluded that the Taylor–Couette UV-C reactors are appropriate for the preservation of a variety of juices, particularly those with high absorption coefficients [131]. Similarly, a study by Orlowska et al. (2014) highlighted that the Taylor–Couette UV unit offered effective mixing that could overcome the limited UV-C penetration depth in opaque beverages like carrot juice [132].

### 5.3. Dean–Vortex-Based Reactors

**Figure 7:**
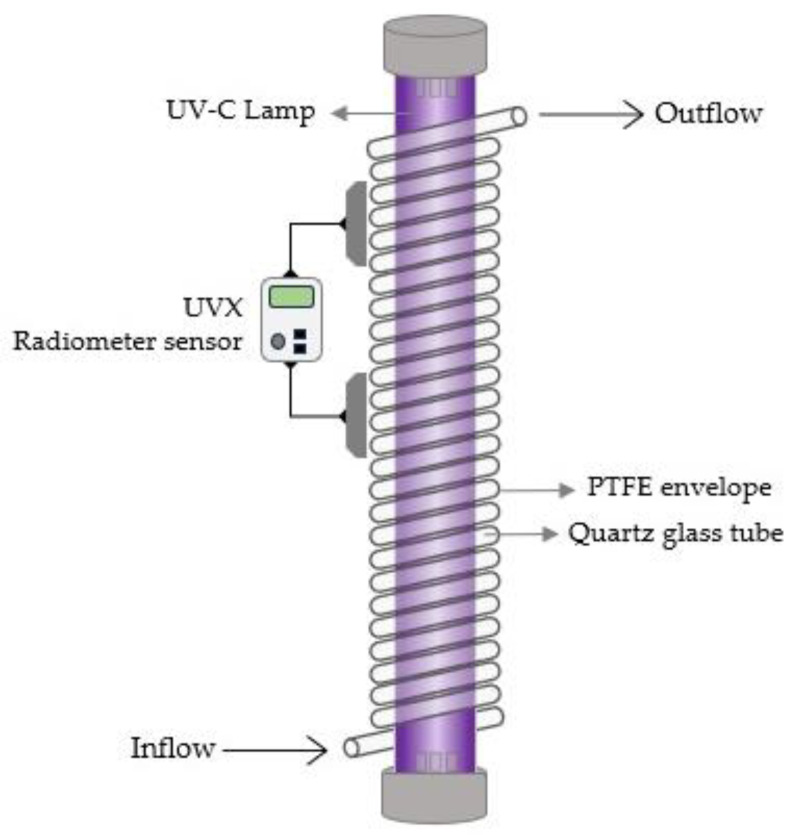
Dean–Vortex UV-C reactor.

A Dean flow system (Figure 7) is characterized by secondary flow vortices (also known as Dean vortices) with the primary forward flow caused by the coiled flow channel in coiled tube reactors. The Dean vortices form as a result of centrifugal forces acting on the fluid volume during rotation. This generates effective radial mixing as well as a greater homogeneity of velocity and residence time distribution (RTD) of the liquid products due to higher Reynold numbers and turbulent conditions, resulting in more uniform treatment conditions [133,134]. The fluid product in these reactors passes through a highly UV-transparent fluorinated ethylene propylene (FEP) tube that is coiled in a helix pattern around one or more UV-C lamps [110]. A Dean flow system consisting of a module made up of a polytetrafluoroethylene (PTFE) envelope with a helically coiled tube tightly fitted to a quartz glass cylinder that houses the UV-C light source was previously used to investigate the formation of toxic compounds in UV-C treated cloudy apple juices. No quantifiable alterations were found in the cytotoxic and genotoxic effects of UV-C treated apple juices [135]. The UVivatec Dean–Vortex reactor was used to treat *Lactobacillus plantarum* BFE 5092 in orange juice. The authors found that increasing the Reynolds number from 86 to 696 led to an increase in the inactivation rate by roughly 2.5 log_10_ cfu/mL [134]. Barut Gök (2021) exposed apple and grape juice to low doses of UV-C in a Dean–Vortex-based reactor. This study demonstrated the potential of this technology to eliminate relevant microorganisms in opaque fruit juice such as *Lactobacillus plantarum* NRIC1749 and *Saccharomyces cerevisiae* NCIB4932 [136]. Orange juice was treated using a modified UV-C reactor based on Dean–Vortex flow. The results indicated that UV-C treatment would be a helpful way to remove or reduce the content of 5-(hydroxymethyl)furfural in orange juice. Additionally, no furan formation was found, and there was no significant alteration in the appearance and color of the juice following UV-C treatment [137]. Cranberry-flavored water was previously treated in a continuous UV-C reactor under a laminar flow regime combined with Dean vortices to ensure suitable mixing, and the treatment enabled a 5-log reduction (99.999%) of *Escherichia coli* ATCC 700728 and *Salmonella enterica* ser. Muenchen ATCC BAA 1764 with a UV-C fluence of 12 mJ·cm^−2^ and 16 mJ·cm^−2^, respectively. In addition, there was no formation of cytotoxic substances up to a UV-C dose of 120 mJ·cm^−2^ [138].

Some examples of commercially available UV-C pasteurizers with their flow types are provided in Table 5.

## 6. Cost Implications, Market Potential, and Consumer Perception

UV-C radiation sources are readily available at affordable costs. This decontamination method is rapid and can be easily integrated into existing food processing systems with low initial investment [142]. The cost of a UV-irradiation unit costs between $10,000–15,000, making it more affordable than a heat pasteurization unit, which costs between $20,000–30,000. The cost of production using UV-C pasteurization may be cheaper than thermal pasteurization. It costs MYR 0.895 ($0.20) to produce UV-C treated pineapple juice in a 320 mL container, while thermally pasteurized pineapple juice costs MYR 0.900 ($0.20) [15]. Similarly, UV-C decontamination costs about MYR 1.60 ($0.35) per 100 L for apple cider, while thermal treatment costs about MYR 4.00 ($0.88). Furthermore, food producers with limited financial resources are likely to benefit from the low initial cost of UV reactors and the low requirement for safety equipment [142].

Compared to other technologies, food and beverage preservation using UV-C is a more sustainable option offering additional cost-saving opportunities due to its reduced machinery cost and electricity consumption. In comparison to heat pasteurization, it has been found that a UV system can consume approximately 10,000 times less energy. Additionally, compared to other emergent food processing technologies like pulsed electric fields (PEF), high-pressure processing (HPP), and membrane filtering (MF), UV also consumes less specific energy when considering an achievement of 5-log reduction in apple juice [143]. The number and kind of UV light sources, the flow rate and pattern, the effectiveness of mixing the UV reactor, and the type/characteristics of the food to be treated (UV light attenuation coefficient, composition of the product, and viscosity) all impact the energy-efficiency of the UV system, and will ultimately influence its operational cost [18].

The operational cost of UV-C food processing is further influenced by a variety of factors including the size/quantity of the food product to be treated, the level of microbial reduction required, the design and capacity of the UV-C equipment, maintenance costs, regulatory compliance, and quality standards. The operational cost of UV-C food processing is between $0.01 and $0.05 per liter for liquid foods and $0.02 to $0.10 per kilogram for solid goods. In the years to come, the market potential for UV-C food processing is anticipated to increase dramatically as consumers desire food products that are fresher, safer, and processed minimally. UV-C is a more sustainable food processing option. The market for UV disinfection equipment was valued at $1.3 billion in 2019 and is anticipated to rise to $5.7 billion by 2027, with a compound annual growth rate (CAGR) of 17.1% from 2020 to 2027. The main factors responsible for propelling the market growth are the environmental and financial benefits of UV-C over conventional technologies and the advent of novel UV-C applications in the food and beverage industries [144]. It is a potential green alternative for processes such as the drying of fresh produce, microbial decontamination of food products (blanching pasteurization and sterilization), post-lethality sanitization of meat, disinfection of food contact surfaces, decontamination of food packaging materials, and extend the shelf life of fresh produce [35,145,146]. However, low-pressure vapor mercury lamps, which are extensively used, present a health risk due to mercury exposure [147].

UV preservation technologies have a good consumer perception due to their numerous benefits including microbial inactivation (including spores) and the deactivation of spoilage enzymes and mycotoxins [59,148]. In a 2022 survey [149], all consumers perceived UV-treated food products as safe for consumption, but they also expressed health-related concerns, primarily due to radiophobia, because numerous consumers still connect radiation with radioactivity and nuclear energy. Furthermore, younger people proved to be more pessimistic, which might be explained by rising health consciousness among this group of respondents, implying that extra efforts will be required for efficient communication to successfully introduce UV-processed foods into the market [149]. The consumer acceptance of UV-treated foods can be increased by omitting the word “radiation” from the label information and adding phrases/terms such as “food safety”, “no radioactivity”, “elimination of microorganisms”, “minimal changes to food”, “absence of residues and toxic effects”, besides the fact that UV preservation is more affordable and energy-efficient than other preservation techniques [149,150].

## 7. International Standards and Regulations for the UV-C Pasteurization of Beverages Foods

UV-C has been used for a long time in the global industry as a viable alternative to thermal pasteurization where its application must guarantee no toxicity to the product, and its use must be allowed by component authorities. The Food and Drug Administration (FDA) approved under regulation 21CFR179.39 the use of UV-C to reduce human pathogens and other microorganisms in juices, control surface microorganisms in food and food products, and the sterilization of water used in food production [151]. The technology is considered germicidal as it effectively inactivates bacteria and viruses with safe use at 253.7 nm, where the preferred UV source for food treatment is low-pressure mercury lamps emitting 90% of emission at a wavelength of 254 nm. However, FDA does not specify any minimum/maximum UV dose levels, where this should be determined on a case-by-case basis, considering good manufacturing practices and situational factors [151]. According to 21CFR179.41, the application of pulsed light technology is approved for food treatment to remove surface microorganisms with a regulated dose below 12 J/cm^2^. The FDA does not have specific regulations or guidance addressed to labeling requirements for foods treated with UV irradiation [152].

The European Union (EU) Novel Food Regulation (Regulation (EU) 2015/2283) is responsible for the authorization and safety assessment of UV-treated foods in the EU as these are considered novel foods [153]. The European Food Safety Authority (EFSA), covered by the regulation in EC no. 258/97, approved the safe use of UV radiation for milk processing post-pasteurization, aiming for the extension of shelf life and to increase the vitamin D content [154]. The intended use of the foods that contain vitamin D resulting from ultraviolet treatment and the specific wavelength ranges allowed for different foods (200–800 nm for mushrooms, 240–315 nm for bread, 200–310 nm for milk, not specified for baker’s yeast) were provided by the European Commission (EC). The EU Regulation also covers the novel foods that need to be authorized before entry into the market in Great Britain since their approach is based on EU processes [30].

The scientific committee of the Food Safety Authority of Ireland published a report in 2020 focusing on the evaluation of emergent food processing technologies (including UV-C) as well as the safety and associated changes in the nutritional content of products treated using emergent technologies in comparison to conventional preservation processes [155]. An evaluation template of novel food processing methods is provided in this report to protect public health and facilitate the development of innovative technologies in the Irish food sector [155]. In Israel, food derived from new production processes like UV-treated milk was approved by The National Food Service at the Ministry of Health to be safely used, where the Israeli food legislation and standardization are under European standards [156]. The regulation requires the product to be labeled as “UV-treated” [30].

In Canada, the Novel Foods Regulation of Health Canada regulates UV-light-treated foods and guarantees the safe use of CiderSure 3500 equipment to achieve at least a 5-log reduction in *E. coli* O157:H7 in unpasteurized and unfermented apple cider and juice [157]. The scientific committee of the Food Safety and Standards Authority of India (FSSAI) has approved raw milk and other dairy products treated with the SurePure UV system with the status “Process Approval” [158].

The knowledge of specific guidelines and regulations regarding UV treatment in each country opens up new opportunities for the further development and commercialization of UV-treated products at an industrial scale.

## 8. Current Limitations and Future Trends of UV-C Food Processing

The biggest obstacle to the commercial use of UV-C technology is its poor penetrating power [159]. The composition and type of food influence UV-C penetration capabilities, being necessary to study foods and beverages case by case to obtain information regarding microbial disinfection and determine the optimal UV-C dosage. This is because the optical and physicochemical characteristics of liquid foods could cause interferences in the target microbe’s exposure to the radiation, which can lessen the efficiency of UV-C radiation [160,161]. For instance, UV-C radiation easily penetrates through transparent or clear liquids. In contrast, because UV-C light can be readily absorbed by opaque matrices, its penetrating potential in opaque foods is quite limited [162]. Additionally, the color and viscosity of the liquid products as well as the presence of natural pigments, organic solutes, and suspended solids limit the penetration of UV photons, lowering the efficacy of UV radiation in inactivating microbes [145,163]. The physicochemical characteristics of treated foods and beverages should be closely monitored because UV-C at high doses may cause the production of hazardous chemicals [164]. Unwanted reactions induced by UV-C can lower vitamin levels, breakdown proteins, degrade antioxidants, oxidize lipids, alter the food color, and generate unpleasant odors [97].

Microbial species can have different degrees of susceptibility and resistance, which can affect the efficacy of UV-C penetration. As such, higher doses of UV-C light will be required for the most resistant species to be inactivated. Because UV-C has a shallow penetration depth, microorganisms need to be directly exposed to it to be inactivated. Hence, a major technical difficulty in commercial UV-C applications is how to guarantee that all the product surfaces are uniformly exposed to UV-C radiation to allow for regular dose delivery and complete microbial exposure [159]. However, altering the flow rate improves its efficiency to inactivate microbes. Another strategy is to employ turbulent flow to guarantee the success of the liquid treatment [124]. Additionally, UV-C can be combined with other low-intensity preservation technologies such as ultrasound, high-pressure processing, and even mild temperatures to enhance overall food safety and quality [165]. However, these combined treatments and hurdle technology should be conducted properly before large-scale implementation to guarantee that the microbiological permissible limits and quality requirements are attained through effective control of the dose and exposure of UV-C [15].

Recently, pulsed ultraviolet (PUV) treatment has been studied for the decontamination of nonsolid foods. PUV has some benefits over continuous UV-C technology: it is rich in UV-C germicidal radiation (200–280 nm) and comprises both the UV-B and UV-A wavelength band (280–400 nm); it has a higher intensity, and shorter treatment duration [30]. Xenon lamps are common light sources in pulsed ultraviolet application, having the advantage of being mercury-free, but their high installation and maintenance costs limit their use in PUV treatments, however, this can be offset by their cheap operating costs and long-lastingness [147]. UV-C radiation from pulsed xenon lamps therefore offers a safe alternative to traditional UV-C food preservation technologies and provides a solution to the low energy output of UV-C radiation through the emission of very high intensity light compared to conventional UV-C light sources such as mercury lamps [25,166]. Nevertheless, the current regulations and guidelines for UV-C application are not standardized in various countries, and information is missing in terms of the exposure times, permissible doses, and labeling requirements, which leads to a variability in the standards, presenting an obstacle for food manufacturers in their food processing operations (see Section 7). Conducting supplementary research and validation studies are a strategic key to optimizing the process and minimizing any negative effects on the safety, sensory quality, physicochemical properties, and nutritional attributes of foods.

## 9. Concluding Remarks

UV-C technology is an environmentally friendly, energy-efficient, and cost-effective process, having appreciable germicidal properties that can inactivate a variety of microbiological pathogens including bacteria, fungi, and viruses to efficiently prevent foodborne diseases and extend the shelf life of food through microbial load reduction without compromising the food’s quality by ensuring minimal alterations to the food’s nutritional value. Numerous food safety applications of UV-C technology exist in the manufacturing of nonsolid foods, ranging from the popular pasteurization of juices to the less common treatment of opaque liquid and semi-liquid plant-based foods, which needs more investigation.

The efficacy of UV-C to preserve nutrients in foods has been discussed extensively, however, the potential contributions of its nutrient retention ability with respect to nutrition insecurity is lacking in the literature. Instances of nutrition insecurity, where individuals do not obtain sufficient nutrients from foods, will inevitably lead to hidden hunger (micronutrient deficiencies). The pasteurization of plant-based nonsolid food has been studied in various UV-C reactor types, and this technology has proven its abilities to reduce the microbial load while causing the minimal degradation of nutrients and health-promoting bioactive compounds in foods. Thanks to this, employing UV-C technology in food production processes can help to tackle hidden hunger and have a positive effect on nutrition security. Consequently, UV-C could be employed as a tool in food design strategies targeting hidden hunger, thereby providing a solution to tackle this global challenge that affects the lives of millions of people.

Although UV-C has limited capacity to penetrate dense and opaque fluids, technological advancements have been made to circumvent the high opacity and high turbidity of some plant-based nonsolid products that lower the UV-C photon penetration such as the use of thin-film systems and the use of equipment that improves flow pattern using turbulent or Dean–Vortex flow [136]. Several difficulties and limitations associated with this technique remain including the need for an adequate reactor design, appropriate selection of processing parameters, and safety precautions. In addition, legislation and standards governing UV-C technology applications in the food industry differ from country to country.

Depending on the specific conditions and requirements of each application, UV-C food processing equipment may incur different operating expenses. A feasibility assessment and a cost–benefit analysis are therefore necessary before introducing UV-C food processing in a food production plant. The use of UV for processing liquid fruit- and vegetable-based foods continues to grow in popularity since it is nonthermal and chemically inert. Although scientists and food professionals know about the advantages and risks of foods processed by UV-C technology, consumers are less informed. An increase in public awareness about this decontamination technology is therefore needed. Additionally, the right labelling can be used to encourage consumers to adopt UV-irradiated food products as rich sources of beneficial nutrients.

## Figures and Tables

**Table 1:** UV-induced microbial inactivation in different types of liquid foods and beverages.

Food Product	Target Microorganism	UV-C Dose (mJ/cm^2^)	Microbial Log Reduction	References
Kale juice	*Escherichia coli* P36	108.3	5.8	[72]
Carrot-orange juice	*Saccharomyces cerevisiae* KE 162	0.016	2.6	[74]
Grape juice	Lactic acid bacteria	78.46	1.6	[75]
Coconut water	*Salmonella typhimurium*	5–30	1.4	[76]
Tomato Juice	*E. coli* O157:H7	191.5	3.83	[77]
Soymilk	*E. coli* W1485 *Bacillus cereus*	11.187	5.6 3.29	[78]
Apple Juice	*E. coli* K12 (ATCC 25253)	707.2	4.4	[79]

**Table 2:** Effects of UV-C light treatment in the quality of health-related bioactive components in fruit- and vegetable-based fluid foods.

Food Product	Bioactive Components	Treatment Conditions	Outcome	References
Apple and pineapple juice	Antioxidant activity Phenolic compounds Vitamin C	UV-C dose: 100–700 mJ·cm^−2^ Exposure time: 5–15 min	Affected the quality of juices, decreasing the bioactive components levels	[89]
Chokanan mango juice (*Mangifera* *indica* L.)	Antioxidant activity Carotenoids Flavonoids Polyphenols	UV-C dose: 3.525 J·m^−2^ Exposure time: 15 and 30 min	UV-C light improved the quality of the juice along with the antioxidant activity and extractability of the bioactive compounds	[90]
Cranberry flavored water	Anthocyanins Ascorbic acid	UV-C dose: 15–240 mJ·cm^−2^ Exposure time: 0 to 403 s	Anthocyanins and ascorbic acid were well retained	[29]
Grape juice (*Vitis labrusca*)	Phenolic compounds	UV-C dose: 65.6 J·m^−2^ Exposure time: 10 min.	Juice from grapes subjected to postharvest UV-C treatment showed an increase in the levels of phenolic compounds	[91]
Grape juice (White “Superior” grape)	Resveratrol phenolic compounds	Irradiation power: 510 W Exposure time: 60 s	UVC treatment enabled the further selective stilbenes enrichment of the juice, especially resveratrol.	[92]
Fresh apple juice	Anthocyanin content Ascorbic acid Antioxidant activity Phenolic compounds Flavonoids	UV-C doses: 84.6–169.1 mJ·cm^−2^ Temperatures: 40, 45, 50, 55, and 60 °C	UV-C irradiation combined with moderate heat treatment increased levels of bioactive compounds	[66]
Lemon Pomace Aqueous Extracts	Antioxidant capacity Phenolic content Flavonoid content Proanthocyanidins	UV-C dose: 4, 19, 80, and 185 kJ·m^−2^ Exposure time: 60, 120, 240, and 360 s	UV-C treatment showed the potential to increase the extraction of bioactive compounds at relatively high dosages.	[93]
Pomegranate juice	Anthocyanins Antioxidant activity Phenolic content	UV-C dose: 12.47 J·mL^−2^, 37.41 J·mL^−2^ and 62.35 J·mL^−2^ Passes: 1, 3 and 5 times	The major quality characteristics of pomegranate juice was better preserved by UV-C treatment than by heating.	[94]
Red Wine (Boğazkere grape)	Anthocyanins Antioxidant activity Phenolic compounds	Thermovinification combined with UV-C	Increased phenolic compounds with health benefits	[95]
Starfruit juice (*Averrhoa carambola* L.)	Flavonols, flavonoids, phenols, antioxidant capacity	UV-C dose: 2.158 J·m^−2^ Exposure time: 0, 30, and 60 min.	UV-C treatment enhanced selected antioxidant compounds	[96]
Strawberry juice	Anthocyanins, ascorbic acid, phenolic compounds	Exposure time: 15–60 min Temperature: 25 ± 1 °C	Decreased the levels of the bioactive compounds	[48]

**Table 3:** Enzyme inactivation studies using ultraviolet light.

Product	Processing Conditions	Results Obtained	Reference
Apple juice	Treatment duration: 2 h Temperature: 25 °C UV source: 400 W high-pressure mercury lamp, 250 to 740 nm	100% inactivation of peroxidase (PO) after 15 min	[101]
Apple juice	Treatment duration: 40 min UV source: UV-LED at 254 nm UV intensity: 0.3 mW/cm^2^ UV dose: 707.2 ± 143.5 mJ/cm^2^	70.43% residual polyphenol oxidase (PPO) activity	[79]
Carrot and orange juice blend	Treatment duration: 1 min UV source: 30 W low-pressure mercury lamp in a tubular reactor Fluence: 10.6 J/cm^2^	18% reduction of pectin methyl-esterase (PME) activity	[102]
Pear juice	Treatment duration: 120 min Temperature: 25 °C UV source: 400 W medium-pressure mercury lamp, 250 to 740 nm	50% reduction in PPO activity after 20 min	[103]
Watermelon juice	Treatment duration: 12 min Temperature: 23 °C UV Source: 9 W UV-C low-pressure mercury lamp at 254 nm Flow rate: 8.4 L/h	35% residual PME activity	[104]
Tomato juice	Treatment duration: 15 min UV source: UV-LED, 278 nm Fluence: 351 mJ/cm^2^	7.31 ± 0.89% residual PME activity	[105]

**Table 4:** UV light in combination with other treatments for juices.

Food Product	Target Microorganism	UV-C Fluence	Combination Technique	References
Tomato Juice	*E. coli* O157:H7 *Salmonella typhimurium* *Listeria monocytogenes*	191.5 mJ cm^2^	Ohmic heating	[77]
Tangerine and grape juices	*S. cerevisiae*	0, 1.64, and 3.13 J m^−l^	Ultrasonic atomization	[111]
Mango juice	Coliform Aerobic bacteria *S. yeasts* Molds	3.525 J m^−1^	Heat (90 °C)	[90]
Carrot juice	*E. coli* O157:H7 *L. monocytogenes STCC 5672*	3.92 J m^−1^	Heat (60 °C)	[112]
Orange juice	*E. coli* O157:H7	0.114 kJ m^−2^	Heat (53 °C)	[113]

**Table 5:** Commercial UV-C food pasteurizers based on different flow patterns.

Equipment Name	Manufacturer	Flow Type	References
Ultraviolet Shockwave Power Reactor (UV-SPR)	Hydrodynamics Inc., Rome, Georgia, USA	Taylor–Couette	[139,140]
UVivatec^®^	Bayer Technology Services GmBH, Leverkusen, Germany	Dean–Vortex	[134]
SurePure Turbulator™	SurePure Inc., New York, USA	Turbulent	[136]
CiderSure UV processor	FPE (Food Processing Equipment, Inc.), Ontario, New York, USA	Laminar	[110]
Aquionics	Hanovia (Nuvonic), Slough, UK	Turbulent	[141]

## Data Availability

Data sharing is not applicable to this article.
